# Synthesis, Characterization, and Antimicrobial Activity of CoO Nanoparticles from a Co (II) Complex Derived from Polyvinyl Alcohol and Aminobenzoic Acid Derivative

**DOI:** 10.1155/2021/6625216

**Published:** 2021-04-27

**Authors:** Maged S. Al-Fakeh, Roaa O. Alsaedi

**Affiliations:** ^1^Department of Chemistry, College of Science, Qassim University, Buraidah 51452, Saudi Arabia; ^2^Taiz University, Taiz, Yemen

## Abstract

Cobalt oxide nanoparticles (CoO NPs) were synthesized by the calcination method from the Co (II) complex which has the formula [Co(PVA)(*P*-ABA)(H_2_O)_3_], PVA = polyvinyl alcohol, and *P*-ABA = *para*-aminobenzoic acid. The calcination temperature was 550°C, and the products were characterized by element analysis, thermal analyses (TGA and DTA), Fourier transform infrared spectroscopy (FTIR), X-ray diffraction (XRD), UV-Vis spectra, and scanning electron microscopy (SEM) techniques. The kinetic and thermodynamic parameters (∆H ^*∗*^, ∆G ^*∗*^, and ∆S ^*∗*^) for the cobalt (II) complex are calculated. The charges been carried by the atoms cause dipole moment 10.53 and 3.84 debye and total energy 11.04 × 10^2^ and 24.80 × 10^2^*k* Cal mol^−1^ for the Co (II) complex and cobalt oxide, respectively. X-ray diffraction confirmed that the resulting oxide was pure single-crystalline CoO nanoparticles. Scanning electron microscopy indicating that the crystallite size of cobalt oxide nanocrystals was in the range of 36–54 nm. Finally, the antimicrobial activity of cobalt oxide nanoparticles was evaluated using four bacterial strains and one fungal strain. Two strains of Gram-positive cocci (*Staphylococcus aureus* and *Enterococcus faecalis*), two strains of Gram-negative bacilli (*Escherichia coli* and *Pseudomonas aeruginosa*), and one strain of yeast such as fungi (*Candida albicans*) were used in this study.

## 1. Introduction

**Figure 1:**
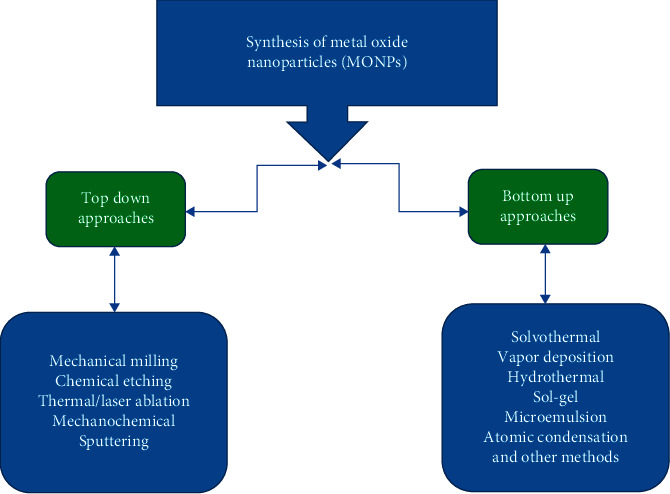
Physicochemical approaches of nanoparticle synthesis.

Recently, metal oxide nanoparticles (MONPs) have been playing a very marked role in many fields of physics, chemistry, and other material science [1, 2]. The transition metals are able to shape a large variety of oxide complexes. Metal oxide nanoparticles (MONPs) exhibit special chemical and physical properties due to their fixed size and more density of the corner. The particle size is likely to be influenced by three important groups of basic properties in more material. The first one involves the structural properties, specifically the lattice symmetry and cell parameter [3–5]. Cobalt oxide nanoparticles have been considerably used in various applications; pigments, rechargeable batteries, catalysts, magnets, sensors, solar energy absorbers, electrochromic devices, and biological activity [6–11]. Usage of cobalt oxide NPs in these applications is based on the physical and chemical properties of this oxide such as crystallinity, composition, crystal size, morphology, and surface area [12]. The physicochemical properties of metal oxide nanoparticles can be planned by synthesis using different methods [13, 14]. The different methods used for synthesis of metal oxides are shown in Figure 1.

The calcination method produced very fine, homogeneous crystalline, high-product-purity, and well-dispersed nanoparticles [15–17]. Polyvinyl alcohol is one of the more important biodegradable, hydrophilic, and semicrystalline polymers, with excellent physical, chemical, and thermal stability. Additionally, polyvinyl alcohol (PVA) is a nontoxic, ecofriendly vinyl polymer soluble in water and highly biocompatible [18–21]. These properties for PVA led to a wide range of applications in pharmaceutical, packaging, medical, cosmetic, and food industries [22]. There are groups that have investigated binary or mixed complexes between polyvinyl alcohol and metal ions [23, 24]. Metal compounds with 4-aminobenzoic acid are among the most explored types of complexes in coordination chemistry and play an important role in catalysis, other materials science, and biochemistry [25, 26]. In this article, preparation and characterization of the cobalt (II) complex containing polyvinyl alcohol and *p*-aminobenzoic acid ligands are described and the resulting complex was used as a raw material for synthesis of nanoparticles of CoO.

## 2. Experimental

### 2.1. Materials and Methods

Polyvinyl alcohol, *para*-aminobenzoic acid, and cobalt (II) chloride hexahydrates supplied by Sigma-Aldrich were used without further purification.

### 2.2. Preparation of [Co(PVA)(*P*-ABA)(H_2_O)_3_]

Polyvinyl alcohol ligand (2 g) was dissolved in 50 mL distilled water by stirring at 50°C for 60 min; after cooling, a CoCl_2_.6H_2_O solution (6.42 g in 35 mL distilled water) was added drop wise to the polyvinyl alcohol solution under quickly stirring, and then, the *P*-aminobenzoic acid solution (3.70 g in 30 mL ethanol) was added to the mixture. Finally, the mixture was refluxed for 3 h and then cooled. The light pink complex was separated by filtration, washed with EtOH, and dried over P_2_O_5_. Anal. Calc. for C_10_H_17_N_2_CoO_7_ : C, 35.73; H, 5.09; N, 8.33. Found: C, 34.96; H, 5.02; N, 8.14. Melting point: 295°C. IR data: *ν* (cm^−1^) = 3420 (m), 3292 (m), 3152 (m), 2922 (m), 2158 (m), 1598 (s), 1539 (s), 1454 (m), 1395 (m), 1084 (s), 880 (m), 845 (m), 782 (m), 618 (m), 590 (m), and 444 (m).

### 2.3. Preparation of Cobalt Oxide Nanoparticles by Calcinations

Direct calcination of the prepared [Co(PVA)(*P*-ABA)(H_2_O)_3_] complex at 550°C with a calcination time of 3 h afforded CoO (NPs). The morphology and size of the as-prepared CoO sample was further investigated using scanning electron microscopy (SEM) and transmission electron microscopy (TEM).

### 2.4. Physical Measurements and Instrumentation

The C, H, and N contents of the solid Co (II) complex were determined by using an Elemental Analyser system GmbhVario El analyzer. FT-IR spectra of the compound and cobalt (II) oxide were obtained by the KBr disc technique; in the wavenumber range of 4000–400 cm^−1^, using a Thermo Nicolet (6700) FT-IR spectrophotometer. The electronic absorption spectral measurements in the ultraviolet and visible regions were carried out in dimethyl sulfoxide on a UV-2102 PC Shimadzu spectrophotometer using a 1 cm matched quartz cell in the wavelength range 200 to 900 nanometer. The magnetic susceptibility of the Co (II) complex was measured at room temperature using a magnetic susceptibility balance of type MSB-Auto. TGA and DTA analyses were completed employing a Shimadzu DTG-60 instrument using a heating rate of 10°C/min in the air atmosphere. The XRD patterns of the CoO NPs were registered using an XRD diffractometer Model (PW 1710) control unit (Philips). The anode material was copper (II) K*α* (*λ* = 1.54180 Å), 40 KV 30 mA; optics: automatic, divergence slit. The scanning electron microscope (SEM) used was JEOL JFC-1100E ION SPUTTERING, DEVICE, JEOL JSM (5400-LV) SEM.

#### 2.4.1. Theoretical Calculations

*(1) Density Functional Theory (DFT)*. Density functional theory (DFT) calculations were accomplished on model complexes derived from the experimental structure data using the Amsterdam Density Functional (ADF) program [27].

#### 2.4.2. Antimicrobial Activity

*(1) Bacterial and Fungal Strains*. The antimicrobial activity of cobalt oxide NPs was evaluated using four bacterial strains and one fungal strain. Two strains of Gram-positive cocci (*Staphylococcus aureus* and *Enterococcus faecalis*), two strains of Gam-negative bacilli (*Escherichia coli,* and *Pseudomonas aeruginosa*), and one stain of yeast such as fungi (*Candida albicans*) were used in this study.

*(2) Inoculum and Cobalt (II) Oxide Nanoparticles Preparation*. Bacterial and fungal inocula were prepared from fresh pure cultures in Muller Hinton broth. Each bacterial and fungal suspension were compared with 0.5 McFarland standard. The CoO nanoparticles solution for antimicrobial activity was prepared by diluting powdered cobalt oxide nanoparticles with 1% DMSO.

*(3) Agar Well Diffusion Assay*. The antimicrobial activity of cobalt oxide nanoparticles against the selected microorganisms was evaluated using the agar well diffusion assay. The bacterial inoculum was spread on the Muller–Hinton agar using a sterile cotton swab by the lawn culture technique. After inoculation, we made 2 wells in the agar plate with the help of the backside blue micropipette tips. Then, cobalt oxide nanoparticle extracts (100 *μ*L) were added to one well and 1% DMSO to another well. Erythromycin_15_, vancomycin_30_, ciprofloxacin_5_, amikacin_30_, and ketoconazole_50_ were used as a positive control. The plates were then incubated for 18–24 hrs at 37°C. After incubation, the zone of inhibition around the wells was observed, and the zone diameter was measured in millimeter (mm) by using a ruler.

## 3. Results and Discussion

**Scheme 1:**
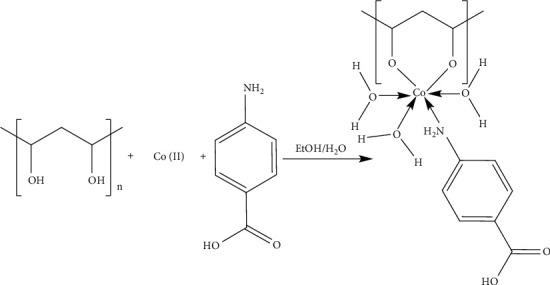
Synthesis of the Co (II) Complex.

The Co (II) complex was prepared by the reaction of polyvinyl alcohol and *p*-aminobenzoic acid in stoichiometric proportions to yield the identical compound according to Scheme 1.

This Co (II) compound was prepared and used as a precursor for cobalt oxide nanoparticles (CoO NPs) by the calcination method. The data correspond to the metal: L1 : L2 ratio of the 1 : 1:1. Also, the complex is air stable, insoluble in common organic solvents, but partially soluble in dimethylsulphoxide (DMSO). The molar conductivity was determined in DMSO with value in the 45 ohm^−1^ cm^2^ mol^−1^ for the Co (II) compound.

### 3.1. FT-IR Spectra

**Figure 2:**
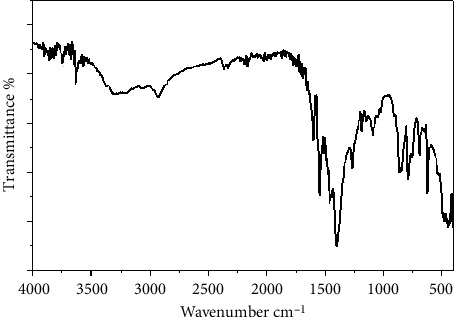
FT-IR of Co (II) complex.

The FT-IR spectrum of the Co (II) complex prepared show an absorption peak was confirmed at *ν* (1178 cm^−1^), and this band has been employed as an estimate polyvinyl alcohol structure because it is a semicrystalline synthetic polymer able to form domains depending on different approach parameters [28]. Additionally, the occurrence of hydrogen-bonded oxygen-hydrogen stretching [29, 30] and antisymmetric stretching vibrations of both CH_2_ and COC at 2921 cm^−1^ and 1138 cm^−1^ for the Co (II) complex, respectively, is found. On the other hand, for polyvinyl alcohol ligand cross linked by the cobalt (II) compound, the band maximum corresponding to the bonded OH group at 3420 cm^−1^, was shifted to lower frequencies, 3292 cm^−1^. The wavenumbers of bands responsible for the amine group in the FT-IR of Co (II) also change in comparison to free *P-*aminobenzoic acid ligand. The (*P-*ABA) ligand displaying a band at 1640 cm^−1^ for the imine *ν* (*C* = *N*) group, which results from the Schiff base condensation of *P*-ABA, was shifted to a lower frequency of 1626 cm^−1^ after complexation [29]. Finally, the appearance of bands at 548 cm^−1^ and 444 cm^−1^ corresponds to *ν* (metal-O) and *ν* (metal-N), respectively [31] (Figure 2).

### 3.2. Electronic Spectra and Magnetic Moments

**Figure 3:**
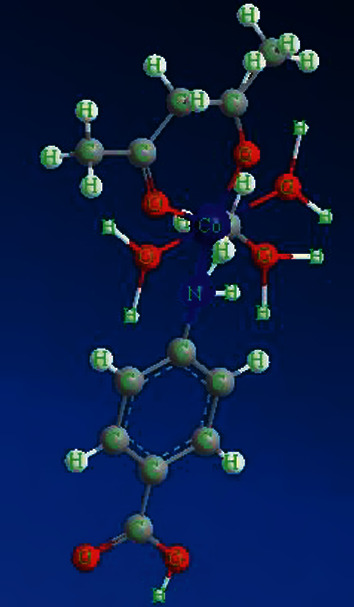
A perspective view of coordination round Co (II) ion.

**Figure 4:**
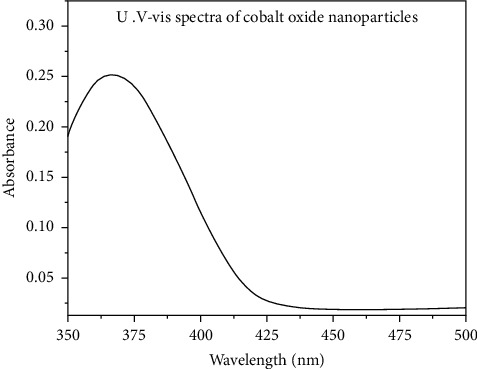
UV-visible image of cobalt (II) oxide nanoparticles.

**Figure 5:**
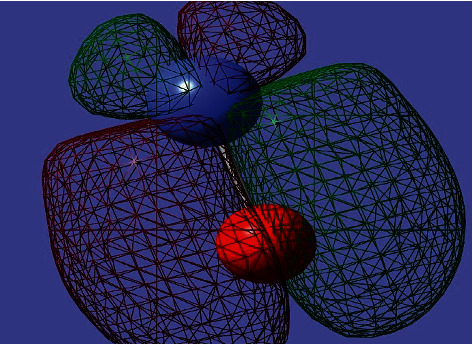
DFT molecular orbital plots for some frontier molecular orbitals of cobalt (II) oxide (HOMO and LUMO).

**Figure 6:**
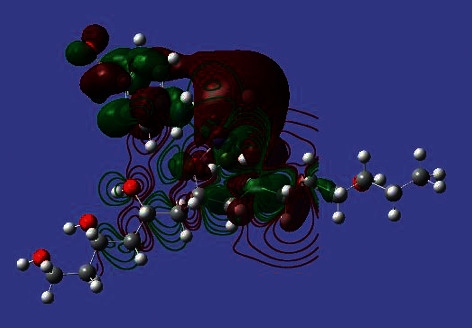
HOMO, LUMO (surfaces), and electron density (contours) for the Co (II) complex.

The electronic spectra of the Co (II) complex and CoO NPs have been recorded in the DMSO solvent. The spectra show two distinct bands in the 32,258 and 26,455 cm^−1^ which corresponds to *π*⟶*π* ^*∗*^ and *n*⟶*π* ^*∗*^ transitions within polyvinyl alcohol and *P*-aminobenzoic acid moieties [32, 33], respectively. There are characteristic bands assigned to the d-d transitions in the Co (II) complex typical of an octahedral structure (Figures 3 and 4). Additionally, the cobalt ((II) compound exhibits a d-d band in 19,230 cm^−1^, and the magnetic moments value was 4.36 BM typical for the octahedral Co (II) complex [34]. The energy of the cobalt (II) complex elucidates that the difference in energy between high occupied molecular orbital (HOMO) and lower unoccupied molecular orbital (LUMO) is called the (HOMO-LUMO) gap; also, this difference in energy between these two boundary orbitals indicated to form the strength and stability of the cobalt (II) complex. DFT MOs in their HOMO-LUMO zone for compounds are displayed in Figures 5 and 6.

### 3.3. Thermal Analysis

**Figure 7:**
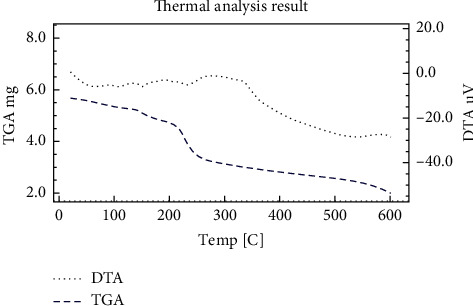
D.T.G and D.T.A curves of the Co (II) complex.

The TG, DTG, and DTA curves (Figure 7) of the Co (II) complex show that the thermal decomposition stages of the compound involve four stages. The four steps occur in the temperature ranges 42–145, 147–205, 207–310, and 312–600°C. The first mass loss correlates well with the release of the three H_2_O molecules (calc. 16.09%, found: 15.92%) with a corresponding DTG peak at 120°C accompanied with a broad endothermic peak in the D.T.A curve at 122°C. The second, third, and fourth steps related to decomposition of the rest of the organic ligands. These steps are marked on the DTG curve at 158, 250, and 548°C with correlating exothermic peaks in the D.T.A curve at 160, 252, and 550°C, respectively. The residue corresponds to a cobalt oxide (calc. 22.29%, found: 20.84%).

#### 3.3.1. Kinetic Analysis and Thermodynamic Parameters for the Co (II) Complex

**Figure 8:**
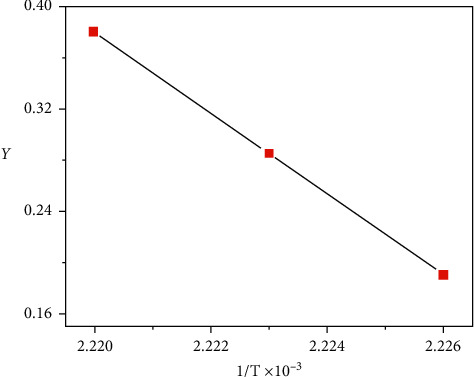
Coats–Redfern plots for the Co (II) complex.

**Figure 9:**
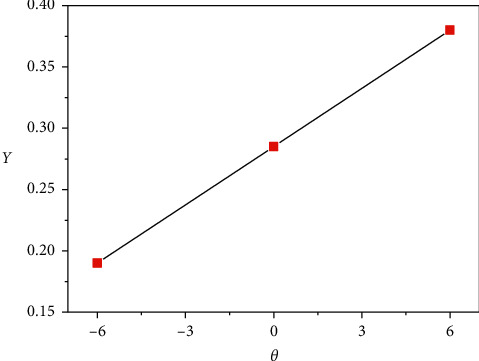
Horowitz–Metzger plots for the Co (II) complex.

Nonisothermal kinetic studies of the Co (II) complex were accomplished applying two different steps: the Coats–Redfern and the Horowitz–Metzger [35, 36] methods (Figures 8 and 9). The kinetic parameters were calculated given to two equations and are listed in Table 1. The activation variables ∆H ^*∗*^, ∆S ^*∗*^, and ∆G ^*∗*^ for the decomposition stages of this compound are recorded in Table 2. Negative ∆S ^*∗*^ numbers of the both steps of decomposition of the Co(II) complex propose that the stimulated compound is highly ordered than the reactants, and the reactions are lower than normal. The positive numbers of (∆G ^*∗*^) denote the decomposition reaction is not spontaneous.

**Figure 10:**
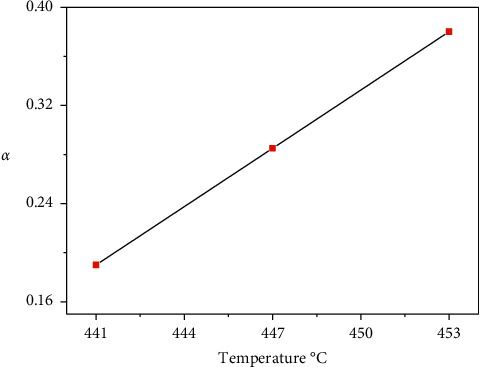
Fraction decomposed (*α*) and temperature plots of the Co (II) complex.

Figure 10 illustrates the relation between *α* and temperature for the Co (II) compound in dynamic air. The figure indicates that the cobalt (II) compound is stable.

### 3.4. X-Ray Powder Diffraction (XRD), SEM, and TEM Analysis

**Figure 11:**
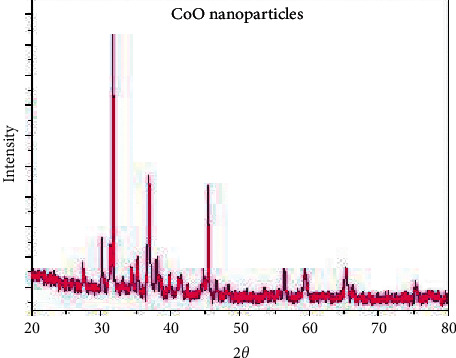
XRD of cobalt oxide nanoparticles.

**Figure 12:**
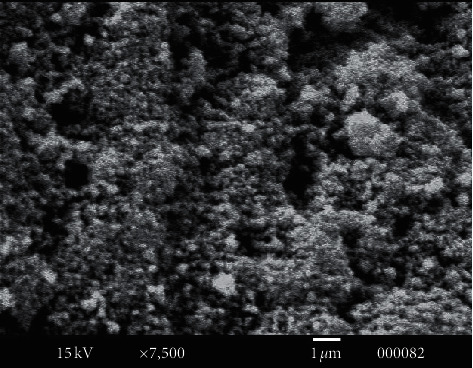
Scanning electron microscope image of cobalt oxide nanoparticles.

**Figure 13:**
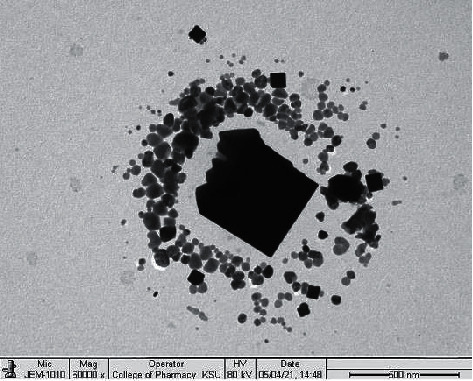
Transmission electron microscope image of cobalt oxide nanoparticles.

XRD patterns of the CoO nanoparticles calcined at 550 °C are shown in Figure 11, which indicates cobalt oxide has a cubic phase structure (Table 3). The average grain size of CoO is determined using the Scherrer relation, and it was found to be around 54 nm.  Figure 12 shows the scanning electron microscopy image where the size of all mesoporous cobalt oxide nanocrystals is distorted growth to near-spherical. On the other hand (Figure 13), as shown in the TEM image, cobalt oxide nanoparticles (with a higher electron density) appear as darker spots. Moreover, the TEM picture shows clearly that the product is entirely composed of crystals with a relatively uniform, separate small spherical and cluster morphology.

### 3.5. Antimicrobial Activity of Cobalt Oxide Nanoparticles

**Figure 14:**
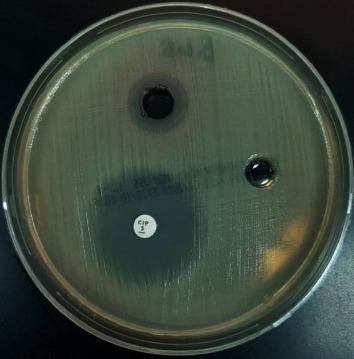
Microbiological screening of the CoO NPs against *E*. *coli*.

**Figure 15:**
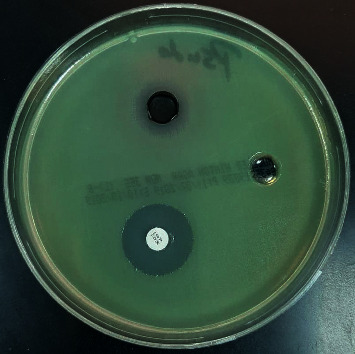
Microbiological screening of the CoO NPs against *Pseudomonas aeruginosa*.

**Figure 16:**
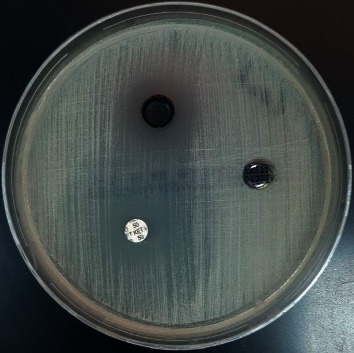
Microbiological screening of the CoO NPs against *Candida albicans*.

Cobalt oxide nanoparticles are well known to have an antimicrobial activity. In this study, it was clear that cobalt oxide nanoparticles had good antimicrobial activity against Gram-negative bacilli and yeast such as fungi, but no activity against Gram-positive cocci. The inhibition zones (mm) for these test organisms are as follows: *Escherichia coli* 16 mm*, Pseudomonas aeruginosa* 15 mm, and *Candida albicans* 20 mm. The big hole is for cobalt (II) oxide nanoparticle, and the others are for the antimicrobial disk and solvent. The microbiological screening of the CoO NPs is shown in Figures 14–16.

## 4. Conclusions

In conclusion, we have demonstrated the facile synthesis of the spherical formed aggregates of cubic-CoO (NPs) by the calcination method of the Co (II) complex. The phase and morphology of the cobalt (II) oxide nanocrystals are characterised by XRD, SEM, and TEM. Results of X-ray sample display that all peaks can be well listed to the phase of CoO. SEM micrographs offer that there are many micropores through the nanocrystals for the sample calcined at 550°C for 3 hours. Also, the antimicrobial activity is tested against standard fungal and bacterial species, and the results present that the calcination method increased antibacterial and antifungal activity than other preparation methods due to small particle size, production of reactive oxygen species (ROS), and large surface area.

## Figures and Tables

**Table 1:** Kinetic parameters for the thermal decomposition of the cobalt (II) complex.

Step	Coats–Redfern equation				Horowitz–Metzger equation			
	*n*	*r*	*E*	*Z*	*n*	*r*	*E*	*Z* x 10^2^
2^nd^	0.00	0.9947	88.1	5.99	0.00	0.9994	93.6	3.51
	0.33	0.9954	93.5	6.36	0.33	0.9958	101.0	7.56
	0.50	0.9955	96.2	6.55	0.50	0.9962	103.8	7.60
	0.66	0.9956	99.2	6.76	0.66	0.9965	106.8	7.64
	1.00	0.9965	105.2	7.18	1.00	0.9970	112.7	7.72
	2.00	0.9979	124.8	8.57	2.00	0.9982	132.2	7.97

E in KJ mol^−1^; underlined *r* in all tables represents the best fit values of *n* and *E*.

**Table 2:** Thermodynamic parameters for the thermal decomposition of the cobalt (II) complex.

Step	*n*	ΔS ^*∗*^	ΔH ^*∗*^	ΔG ^*∗*^
2^nd^	0.00	−203.51	89.97	180.93
	0.33	−197.13	97.32	185.43
	0.50	−197.09	100.13	188.22
	0.66	−197.05	103.10	191.18
	1.00	−196.96	109.06	197.10
	2.00	−196.70	128.57	216.49

∆H ^*∗*^ and ∆G ^*∗*^ in kJ mol^−1^ and ∆S ^*∗*^ in kJ mol^−1^K^−1^.

**Table 3:** XRD crystal data of the CoO NPs.

Parameters	CoO
Empirical formula	CoO
Formula weight	74.92
Crystal system	Cubic
a (Å)	8.08
b (Å)	8.08
c (Å)	8.08
*α* (°)	90.00
*β* (°)	90.00
*γ* (°)	90.00
Volume of the unit cell (Å3)	528.12
Particle size (nm)	54

## Data Availability

The data used to support the findings of this study are available within the article.
